# High-Fat Diet Alters the Retinal Transcriptome in the Absence of Gut Microbiota

**DOI:** 10.3390/cells10082119

**Published:** 2021-08-18

**Authors:** David Dao, Bingqing Xie, Urooba Nadeem, Jason Xiao, Asad Movahedan, Mark D’Souza, Vanessa Leone, Seenu M. Hariprasad, Eugene B. Chang, Dinanath Sulakhe, Dimitra Skondra

**Affiliations:** 1 Department of Ophthalmology and Visual Science, University of Chicago, Chicago, IL 60637, USA; David.Dao@uchospitals.edu (D.D.); jason.xiao@uchospitals.edu (J.X.); sharipra@bsd.uchicago.edu (S.M.H.); 2 Center for Research Informatics, University of Chicago, Chicago, IL 60637, USA; bxie@medicine.bsd.uchicago.edu (B.X.); dsouza@bsd.uchicago.edu (M.D.); 3 Department of Medicine, University of Chicago, Chicago, IL 60637, USA; sulakhe@uchicago.edu; 4 Department of Pathology, University of Chicago, Chicago, IL 60637, USA; Urooba.Nadeem@uchospitals.edu; 5 Department of Ophthalmology and Visual Science, Yale University School of Medicine, New Haven, CT 06437, USA; asadolah.movahedan@yale.edu; 6 Department of Animal Biologics and Metabolism, University of Wisconsin, Madison, WI 53706, USA; valeone@wisc.edu; 7 Knapp Center for Biomedical Discovery, Department of Medicine, Microbiome Medicine Program, University of Chicago, Chicago, IL 60637, USA; echang@medicine.bsd.uchicago.edu

**Keywords:** age-related macular degeneration, high-fat diet, gut microbiome, gut-retina axis, RNA sequencing, germ-free mice, complement cascade, angiogenesis, retinal inflammation

## Abstract

The relationship between retinal disease, diet, and the gut microbiome has shown increasing importance over recent years. In particular, high-fat diets (HFDs) are associated with development and progression of several retinal diseases, including age-related macular degeneration (AMD) and diabetic retinopathy. However, the complex, overlapping interactions between diet, gut microbiome, and retinal homeostasis are poorly understood. Using high-throughput RNA-sequencing (RNA-seq) of whole retinas, we compare the retinal transcriptome from germ-free (GF) mice on a regular diet (ND) and HFD to investigate transcriptomic changes without influence of gut microbiome. After correction of raw data, 53 differentially expressed genes (DEGs) were identified, of which 19 were upregulated and 34 were downregulated in GF-HFD mice. Key genes involved in retinal inflammation, angiogenesis, and RPE function were identified. Enrichment analysis revealed that the top 3 biological processes affected were regulation of blood vessel diameter, inflammatory response, and negative regulation of endopeptidase. Molecular functions altered include endopeptidase inhibitor activity, protease binding, and cysteine-type endopeptidase inhibitor activity. Human and mouse pathway analysis revealed that the complement and coagulation cascades are significantly affected by HFD. This study demonstrates novel data that diet can directly modulate the retinal transcriptome independently of the gut microbiome.

## 1. Introduction

Over the last several decades, there is increasing evidence that diet and nutrient intake contribute to the pathophysiology of retinal diseases, including age-related macular degeneration (AMD), diabetic retinopathy (DR), and glaucoma [1,2,3,4]. The retina is one of the most metabolically active tissues in the body, and with its rich store of polyunsaturated fats, is vulnerable to oxidative, metabolic, and fatty acid perturbances [5,6]. In particular, multiple research groups have linked high-fat diets (HFDs) and fat-specific intake with increased prevalence of intermediate or advanced AMD, the leading cause of blindness in the developed world [7,8,9,10]. HFDs have been shown to replicate or exacerbate features of retinal disease through various proposed mechanisms: lipid signaling, metabolic dysfunction, vascularization, and inflammatory regulation [11]. Compared to mice fed on conventional or low-fat diets, HFD-fed mice exhibit impaired retinal sensitivity, greater macrophage/microglial cell activation, altered retinal fatty acid composition, and hallmark features of AMD such as choroidal neovascularization (CNV) and sub-retinal pigment epithelium (RPE) deposits [12,13,14,15]. HFDs also can lead to systemic changes such as hypercholesteremia and hyperinsulinemia, paving the way for retinal disease risk factors like obesity and diabetes [16]. HFD-induced vascular changes in the eye have also been reported, including changes in permeability and formation of acellular capillaries, though these effects are not consistent across studies [16,17]. Use of electroretinograms (ERGs) support the notion that HFDs can negatively affect function of several retinal cell types, including photoreceptors, bipolar cells, and retinal ganglion cells [16]. Altogether, a small but growing body of literature suggests that HFDs alter the retina and its microenvironment.

Recent studies suggest that the effects of HFDs in retinal diseases may be in part mediated through the gut microbiome [18]. The human gut microbiome is comprised of trillions of microbiota that live within our gastrointestinal tracts. These microbes can maintain and alter health homeostasis, playing diverse roles in immune regulation, metabolism, drug processing, and intercellular signaling [19]. This rapidly growing body of literature has linked the gut microbiome with anatomically distant sites, including the heart, liver, brain, and lungs [20,21,22,23]. Research regarding the gut microbiome’s role in ocular tissues, particularly in the retina, has only recently begun [24,25]. These pioneering studies have revealed functional and compositional differences in the gut microbiome in patients with retinal diseases such as primary open-angle glaucoma (POAG), neovascular AMD, retinal artery occlusion (RAO), and retinopathy of prematurity (ROP) [26,27,28,29]. Our team has previously shown that modulating the gut microbiome impacts the retinal transcriptome across many biological pathways implicated in retinal disease [30]. Other investigators have found that gut microbiota can alter retinal lipid composition, as well as systemic factors such as endotoxemia and immune response that may set the stage for retinal pathophysiology [31,32]. Due to their location in the gastrointestinal system and intimate communication with human cell types, gut microbiota may be responsible for mediating many of the observed effects of HFD on the retina. For example, Andriessen et al. recently showed that a HFD modifies gut microbiota composition, which consequently exacerbates laser-induced CNV [18]. Other studies have also shown that HFD-fed mice exhibit altered gut microbiomes, such as reduced diversity and a decreased Bacteroidetes-to-Firmicutes ratio [33,34,35].

Taken together, there seems to be a connection between HFD, the gut microbiome, and retinal homeostasis. Nevertheless, whether and how HFD affects the retina in the absence of gut microbiome remain unknown. In this study, we sought to distinguish these overlapping components, and investigate whether HFD directly alters the retinal transcriptome in the absence of gut microbiome by using germ-free mice and high-throughput RNA-sequencing. Given the complexity of genetic and environmental factors that contribute to retinal disease progression, understanding how diet impacts retinal biology at the transcriptional level could help identify underlying mechanisms of how diet can affect retinal diseases, which ultimately could lead to discovery of novel targets for preventative or therapeutic interventions to treat ocular diseases.

## 2. Materials and Methods

### 2.1. Animals and Diets

Mouse experiments were approved by the University of Chicago Institutional Animal Care and Use Committee and conducted according to ophthalmic and vision research guidelines set by the Association for Research in Vision and Ophthalmology (ARVO). This study used germ-free (GF) C57B1/6 adult male mice, which were housed in the Gnotobiotic Research Animal Facility at the University of Chicago. At 7 weeks of age, GF mice were fed ad libitum either a normal diet (ND) or high-fat diet (HFD) for 8 consecutive weeks. The HFD consisted of 23% saturated fat. Environmental conditions, including humidity and temperature, adhered to The Guide for the Care and Use of Laboratory Animals, 8th edition, and mice were subjected to a standard 12-h light cycle. At 15 weeks of age, the mice were euthanized by carbon dioxide and subsequent cervical dislocation. Samples were immediately placed on ice and processed for RNA-sequencing.

### 2.2. Sterility Monitoring

In order to provide a sterile environment, GF mice were housed in positive-pressure incubators at the University of Chicago’s Gnotobiotic Research Animal Facility and fed diets which had been irradiated and autoclaved at 250 °F for 30 min. Germ-free status was assessed as described previously [36]. Briefly, fecal samples were collected weekly, and cultured aerobically at 37 °C and 42 °C and anaerobically at 37 °C. Cultures were checked after 1, 2, 3, and 5 days had passed—no positive cultures were identified during the study. Additionally, fecal samples were screened for contamination by DNA extraction and quantitative real-time polymerase chain reaction (PCR) using universal bacterial primers for the 16 S RNA-encoding gene (IDT, 8 F was 5′-AGA GTT TGA TCC TGG CTC AG-3′, and 338 R was 5′-TGC TGC CTC CCG TAG GAG T-3′).

### 2.3. RNA Extraction

Whole mouse retinas were isolated on ice from freshly enucleated eyes, with all equipment, surfaces, and tubes treated with RNase decontamination solution (Thermo Fisher Scientific, Waltham, MA, USA) prior to use. Dissected retinas were stored in RNAlater solution (Thermo Fisher Scientific, Waltham, MA, USA) at −80 °C until RNA extraction using the RNeasy kit from Qiagen (Qiagen, Hilden, Germany). Concentrations were quantified using a Nanodrop (Thermo Fisher Scientific, Waltham, MA, USA) before sequencing.

### 2.4. RNA Sequencing

RNA from eight samples was used for analysis (four per diet group). The quality was evaluated using a Bioanalyzer at the University of Chicago Genomics Core and was confirmed to meet appropriate RNA integrity numbers (RIN). Next, cDNA libraries were constructed using TruSeq RNA Sample Prep kits (Illumina, San Diego, CA, USA) to generate 100-bp paired-end reads, which were indexed for multiplexing and then sequenced using PE100bp on the NovaSeq 6000 System (Illumina, San Diego, CA, USA). Data was provided in FASTQ format and analyzed in R.

### 2.5. Statistical Analysis

The secondary analysis of sequence data was performed on Globus Genomics, an enhanced, cloud-based analytical platform that provides access to different versions of Next-Generation Sequence analysis tools and workflow capabilities. Tools such as STAR, featureCounts, and Limma were run from within the Globus Genomics platform. We used STAR (version 2.4.2 a, Stanford University, Stanford, CA, USA) aligner default parameters to align the RNA-seq reads to the reference mouse genome (GRCm38) for all eight samples. The raw gene expression count matrix was then generated by featureCounts (version subread-1.4.6-p1). The gene annotation was obtained from the Gencode vM23. STAR default parameter for the maximum mismatches is 10 which is optimized based on mammalian genomes and recent RNA-seq data.

Significant DEGs with a *p*-value < 0.01 and LogFC > 1 were extracted for further downstream analysis. Filtering for DEGs with low expression (count-per-million < 10) was performed using edgeR [37,38]. The enrichment analysis in EnrichR suite took both the upregulated and downregulated DEGs in GF and extracted the over-represented gene ontology functional classification (molecular functions, biological processes, and cellular component). The significance of the association between the datasets and bio functions were measured using a ratio of the number of genes from the dataset that map to the pathway divided by the total number of genes in that pathway. This enrichment analysis was based on mouse-to-human orthologs. A list of all DEGs and their *p*-values is available in Table 1 and Table 2.

## 3. Results

### 3.1. HFD Is Associated with Differential Retinal Gene Expression in the Absence of the Microbiome

**Figure 1:**
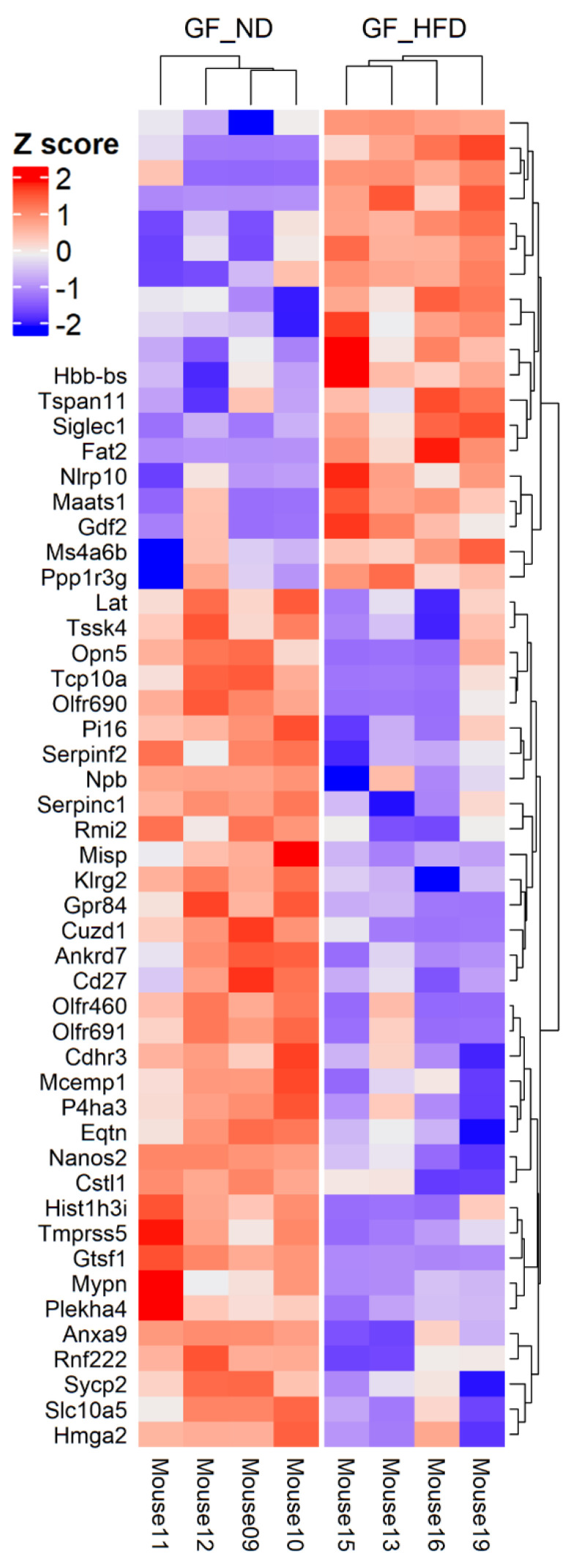
Heatmap with hierarchal clustering demonstrating 53 differentially expressed genes (DEGs) with a logFC greater than 1 and *p*-value < 0.01 between germ-free mice on normal diet (GF-ND, *n* = 4) and germ-free mice on high-fat diet (GF-HFD, *n* = 4). Z-score is calculated from LogFC with red and blue indicating upregulated and downregulated genes, respectively.

To compare the effect of a high-fat diet on the retinal transcriptome, we performed high-throughput RNA-seq analysis of mouse retinas from the GF-ND and GF-HFD. We sequenced four whole retinas from both experimental groups (*n* = 4 eyes from 4 different mice, controlled for age and sex). After the correction of the raw data to remove background noise, 19,681 genes were selected for differential gene analysis (Supplementary Table S1). DEGs were selected based on a stringent *p*-value cutoff < 0.01 and logFC > 1. Comparison between the two groups identifies 53 DEGs, of which 19 are upregulated and 34 are downregulated in the GF-HFD mice group. The National Center for Biotechnology Information (NCBI) gene database was used to filter pseudogenes and uncharacterized cDNA to compile a list of protein-coding genes only. A heatmap was plotted to show the hierarchical clustering of the DEGs (Figure 1). The sequencing data suggests that HFD is associated with changes in the retinal transcriptome in the absence of the microbiome. Detailed list and statistics of the upregulated and downregulated DEGs are available in Table 1 and Table 2.

### 3.2. Significant Biologic Functions and Processes Are Overrepresented by Functional Enrichment Analysis

**Figure 2:**
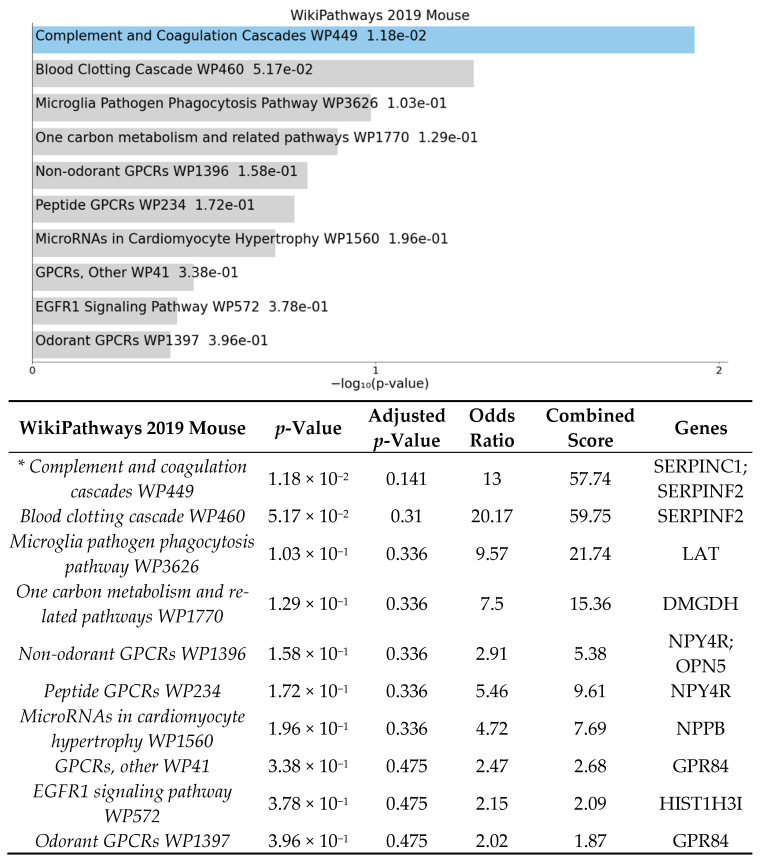
Gene list enrichment analysis of significant DEGs between GF-HFD mice (*n* = 4) and GF-ND mice (*n* = 4) using EnrichR. The bar graph shows a ranked list by *p*-value of the top 10 over-represented mouse pathways with significant pathways indicated in blue (*p*-value < 0.05). The corresponding table demonstrates detailed statistics and involved genes with significant pathways indicated by an asterisk (*p*-value < 0.05).

**Figure 3:**
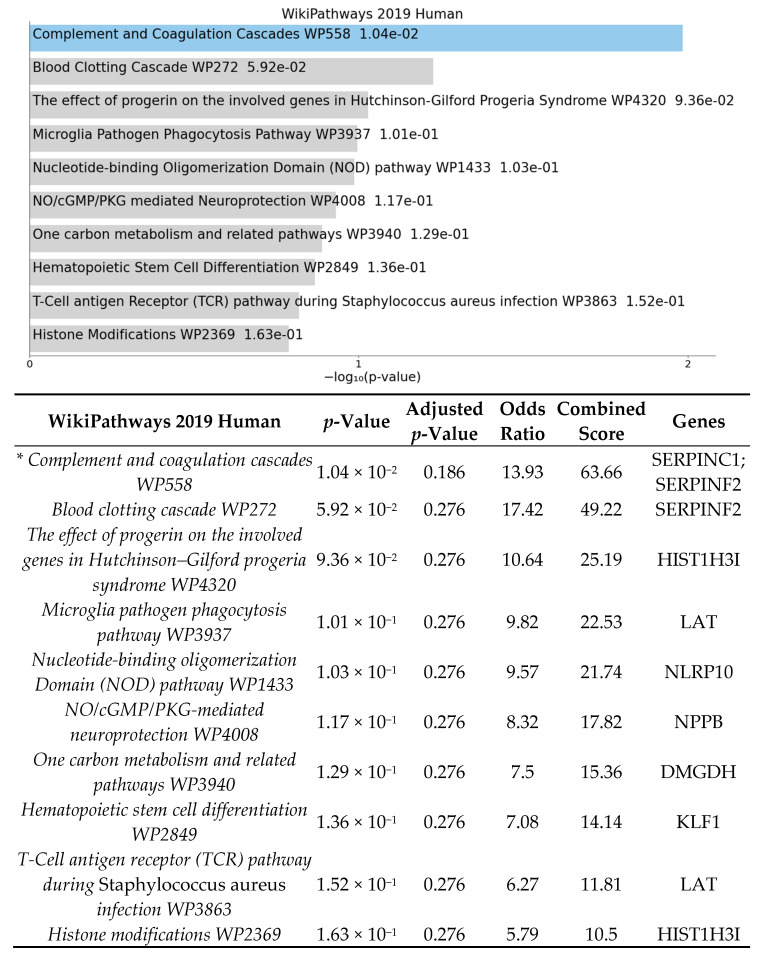
Gene list enrichment analysis of significant DEGs between GF-HFD mice (*n* = 4) and GF-ND mice (*n* = 4) using EnrichR. The bar graph shows a ranked list by *p*-value of the top 10 over-represented human pathways with significant pathways indicated in blue (*p*-value < 0.05). The corresponding table demonstrates detailed statistics and involved genes with significant pathways indicated by an asterisk (*p*-value < 0.05).

**Figure 4:**
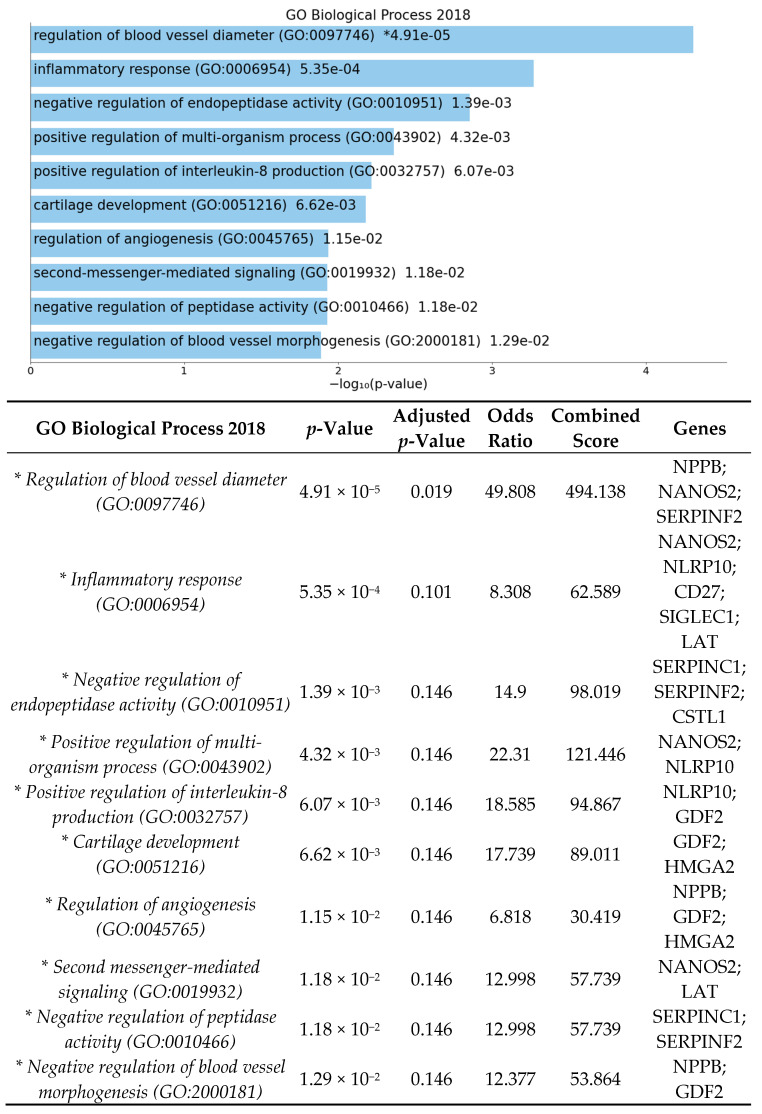
Gene list enrichment analysis of significant DEGs between GF-HFD mice (*n* = 4) and GF-ND mice (*n* = 4) using EnrichR. The bar graph shows a ranked list by *p*-value of the top 10 over-represented biological processes with significant processes indicated in blue (*p*-value < 0.05). The corresponding table demonstrates detailed statistics and involved genes with significant processes indicated by an asterisk (*p*-value < 0.05).

**Figure 5:**
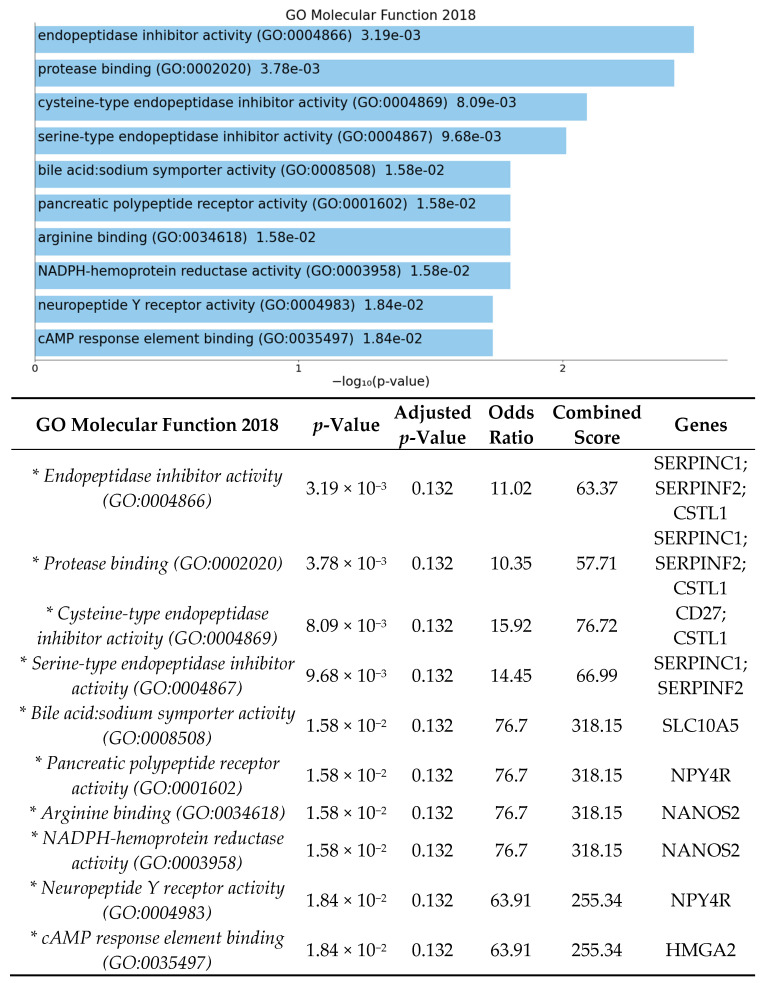
Gene list enrichment analysis of significant DEGs between GF-HFD mice (*n* = 4) and GF-ND mice (*n* = 4) using EnrichR. The bar graph shows a ranked list by *p*-value of the top 10 over-represented molecular functions with significant functions indicated in blue (*p*-value < 0.05). The corresponding table demonstrates detailed statistics and involved genes with significant functions indicated by an asterisk (*p*-value < 0.05).

The enrichment analysis for gene ontology and pathways was performed using EnrichR [39,40,41]. Enrichment analysis was done to identify over-represented biological functions and classes from statistically significant differentially expressed genes. Human and mouse pathway analysis revealed that complement and coagulation cascades were significantly affected by HFD (Figure 2 and Figure 3). The analysis also shows that the top 3 biological processes are regulation of blood vessel diameter, inflammatory response, and negative regulation of endopeptidase (Figure 4). Molecular functions altered include endopeptidase inhibitor activity, protease binding, and cysteine-type endopeptidase inhibitor activity (Figure 5).

## 4. Discussion

To our knowledge, this is the first study to use high-throughput RNA sequencing of whole retinas from GF mice to demonstrate that high-fat diet alone is associated with changes in the retinal transcriptome in the absence of gut microbiome. Diet is a highly modifiable risk factor for development of vision threatening diseases, and understanding the relationship between diet and ocular pathology is a promising avenue for intervention. However, the biological pathways connecting diet to ocular disease are poorly understood and there is limited literature investigating the pathways involved.

Multiple clinical studies have demonstrated that diet plays a critical role in retinal health and contributes to diseases including age-related macular degeneration, diabetic retinopathy, and primary open angle glaucoma [42,43,44]. For example, recently published data from the AREDS study group reported that higher intake of saturated fatty acids, monounsaturated fatty acids, and oleic acid were associated with significant increased risk of progression to late AMD [45]. Supporting this, our team has previously published data showing that high-fat diet increased lesion size, vascular leakage, and sub-RPE deposits of laser-induced choroidal neovascularization in both wild-type and apolipoprotein E-deficient mice [15]. Recent evidence has suggested that the effects of high-fat diet on retinal disease are mediated by the gut microbiome. High-fat diets can cause gut microbial dysbiosis altering intestinal permeability and leading to low-grade inflammation with release of pro-angiogenic factors which may exacerbate ocular diseases such as proliferative diabetic retinopathy and neovascular AMD [18].

To further elucidate the biological connections between the diet–gut–retina axis, we aimed to investigate how diet affects the retinal transcriptome independently of the gut microbiome [30]. Germ-free mice, raised without exposure to any microbes, provide an ideal model to investigate this hypothesis [46]. In this study, we used GF mice fed a high-fat diet compared to a normal diet to explore retinal transcriptome changes induced by diet alone. After analysis of 19,681 total DEGs with removal of pseudogenes, 53 significant DEGs with LogFC > 1 were identified between groups (Figure 1). Enrichment analysis shows pathways involved in complement and coagulation cascades, inflammatory response, regulation of angiogenesis and blood vessel morphology, and endopeptidase inhibitor activity (Figure 2) were significantly affected by high-fat diet in germ-free mice.

### 4.1. High-Fat Diet May Affect Expression of Genes Involved in Inflammatory Pathways in Germ-Free Mice

Enrichment analysis of significant DEGs demonstrated that complement and coagulation cascades were significantly affected by high-fat diet (Figure 2). The complement and coagulation cascades are activated in response to retinal inflammation and vascular injury and have been highly implicated in retinal disease, especially in development of age-related macular degeneration, with multiple ongoing clinical trials currently being investigated [47,48,49]. Additional biological pathways identified were involved in inflammatory response, positive regulation of interleukin-8, protease binding, and regulation of endopeptidase activity (Figure 4 and Figure 5). Our results demonstrate that DEGs in pathways involved in retinal inflammation were significantly affected by high-fat diet (Table 1 and Table 2). *C1qtnf2* is a member of the C1q and tumor necrosis factor related-protein (CTRP) superfamily reported to be involved in retinal inflammation and associated with late-onset retinal degeneration [50,51]. High expression of CTRPs has been reported in the drusen of human donor eyes with AMD [52]. Additionally, the CTRP family has reported to mediate glucose-induced oxidative stress and apoptosis in RPE cells [53]. *Ifi204* (interferon gamma inducible protein) is a cytosolic DNA sensor involved in initiation of a type 1 interferon response and activation of the inflammasome pathway in response to bacterial or viral infection [54,55]. Multiple genes involved in activation of local ocular inflammatory response, including *Ifi204*, have been identified as mediators of retinal aging [56,57]. The H3 family of histones (including *Hist1h3i*) may be important in epigenetic modifications that promote a persistent pro-inflammatory state in diabetic retinopathy [58,59]. Multiple classes of histone genes are involved in regulation of the nucleosome and have been shown to be actively transcribed in both developing and aging retinal neurons [60]. *Serpinc1* and *Serpinf2* are members of the serine protease inhibitor (serpin) family, which were also found to be downregulated in our study. Proteins in the serpin family include endopeptidases that have been reported to be important in inhibiting angiogenesis and retinal cell death [61,62]. Proteomics analysis has identified multiple proteins in the serpin family including both *Serpinc1* and *Serpinf2* as potential serum biomarkers of retinal inflammation in diabetic retinopathy [63]. Additionally, VEGF is involved in the negative regulation of cysteine-type endopeptidase activity required for the apoptotic process [64].

### 4.2. High-Fat Diet May Influence Genes and Pathways Involved in Angiogenesis in Germ-Free Mice

Enrichment pathway analysis of the significant DEGs showed that pathways involved in regulation of angiogenesis, blood vessel diameter, and blood vessel morphogenesis (Figure 4) were affected by high-fat diet in germ-free mice. Bioactive lipids have been shown to be involved in regulation of pathologic retinal angiogenesis [65]. Our results identified several DEGs involved in regulation of angiogenesis (Table 1 and Table 2). *Fat2* (FAT-like cadherin 2) was the most highly upregulated gene identified and has not previously been described in the retina [66]. The cadherin superfamily is involved in maintaining the blood–retinal barrier and cell migration during angiogenesis [67,68]. *Neuropsin* (Opn5) is expressed in retinal ganglion cells and has to been reported to mediate light-dependent retinal vascular development and mediate photoentrainment of circadian rhythm [69,70]. *Nppb* (Natriuretic peptide B) is involved in retinal response to hypoxia and may be an important regular of retinal vascular permeability [71,72].

### 4.3. High-Fat Diet May Affect Expression of Genes Involved in RPE Function and Ciliogenesis in Germ-Free Mice

Our data also suggest that high-fat diet may regulate expression of several genes involved RPE function and ciliogenesis in germ-free mice. Multiple DEGs related to olfactory receptor expression in the mouse retina (*Olfr460*, *Olfr690*, and *Olfr691*) were found to be affected by high-fat diet. Recent literature using RNA-sequencing of human retina has demonstrated that olfactory receptors are expressed in human retina in the retinal pigment epithelium, photoreceptor inner segments, ganglion cell layer, bipolar cells, and horizontal cells [73]. Retinal olfactory receptors may be important in retinal repair involving retinal pigment epithelium and retinal neurons [74,75]. Olfactory receptor expression is hypothesized to induce RPE proliferation and migration [76]. *Gtsf1* (gametocyte specific factor 1) was also identified as highly downregulated by high-fat diet in the retina of germ-free mice and has not previously been reported to be expressed in the retina. *Gtsf1* is involved in retrotransposon suppression in germ cells to prevent genomic instability [77]. Retrotransposons are also reported to be involved in propagation of *Alu* retroelements which may contribute to RPE cell death in age-related macular degeneration [78]. *Cstl1* (cystatin-like 1) was also identified to be downregulated by high-fat diet; however, the specific function of *Cstl1* is currently unknown. Other members of the cystatin superfamily, notably cystatin C, are highly expressed in the RPE and are associated with increased risk of AMD and Alzheimer’s disease [79,80,81,82]. *Deup1* (deuterostome assembly protein 1) is an important component of the deuterosome involved in ciliogenesis [83]. While the exact role of Deup1 in the retina has not been determined, defects in primary cilium function in photoreceptors and the RPE leads to retinal degeneration as part of several syndromic ciliopathies like Bardet-Beidl syndrome and Alstrom syndrome [84]. *Maats1* (Cilia and Flagella associated protein 91) has been identified as an important component of sperm flagellum structure and has not previously been described in the retina [85]. Mutations in the cilia and flagella-associated protein family have been linked with retinitis pigmentosa in familial amyotrophic lateral sclerosis [86].

### 4.4. Additional Genes and Pathways of Retinal Transcriptome Affected by High-Fat Diet in Germ-Free Mice

Several neuroendocrine related pathways including pancreatic polypeptide receptor activity and neuropeptide Y receptor activity were found to be affected by high-fat diet in germ-free mice.

*Npy4r* (neuropeptide Y receptor) is expressed in human retinal RPE and glial cells, and it is involved in neuronal calcium release, neuroprotection, and proliferation of glial cells [87]. Clinically, polymorphisms in NPY have been associated with increased risk of type 2 diabetes and development of diabetic retinopathy [88,89]. Neuropeptide b (*Npb*) is a relatively novel neuropeptide associated with regulation of the neuroendocrine system, pain processing, stress, and feeding behaviors [90]. *Npb* is widely expressed in the central nervous system, but expression has not previously been described in the retina [91].

Several identified significant DEGs have not previously been reported to be expressed in the retina. *Rmi2* is involved in genome stability and has been reported to be associated with development of multiple types of cancer [92,93,94]. The physiologic role of *Rnf222* has not been described currently; however, other members of the ring finger protein family have been associated with cerebral vascular diseases like Moyamoya disease and atherosclerotic stroke [95]. *Cuzd1* (CUB and zona pellucida-like domains 1) has been reported to mediate epithelial proliferation of the mammary gland during pregnancy [96]. *Cuzd1* has also been identified in human embryonic stems cells [97]. Single nucleotide polymorphisms (SNPs) in *Cuzd1* have been associated with risk of age-related macular degeneration [98]. *Dmgdh* (dimethylglycine dehydrogenase) is involved in choline metabolism important in neurotransmitter and phospholipid biosynthesis [99]. *Dmgdh* was identified as part of a set of differentially expressed genes in the mitochondrial transcriptome human retinas with diabetic retinopathy [100]. *Nanos2* (nanos C2HC-type zinc finger 2) is involved in germ cell differentiation and was also identified as differentially expressed in the retinal transcriptome of a mouse model of diabetic retinopathy [101].

## 5. Conclusions and Limitations

This study demonstrates novel data that suggest diet may modulate the retinal transcriptome in the absence of the gut microbiome. Unbiased analysis of the retinal transcriptome using high-throughput RNA-sequencing identified genes and pathways involved in retinal inflammation, angiogenesis, and RPE function, whose expression was influenced by HFD in the absence of gut microbiome. These genes and pathways may be involved in complex diet-microbiome-retina axis interactions that have only recently been recognized to play roles in retinal physiology and retinal disease pathogenesis.

Our study is limited to RNA-sequencing alone, and confirmatory studies of protein expression or activity were outside the scope of this investigation. The goal of our study was to use germ-free mice and RNA-sequencing technology to provide an unbiased characterization of the effects of HFD on global retinal gene expression in the absence of gut microbiome, as well as to identify potential novel targets within the retinal transcriptome that may guide future investigation on the diet-microbiome-retina axis.

Future studies with quantitative RT-PCR, proteomics, or functional assays are needed to further investigate potential functional pathways affected by HFD. In addition, studies using animal models of retinal diseases should include protein markers of angiogenesis and retinal apoptosis using multiplex assays, ELISA, Western blotting, or flow cytometry to better characterize how the genes and pathways revealed by high-throughput RNA-sequencing may be modulated by HFD. Applying a multiomics approach towards investigating the diet-microbiome-retina axis will be critical to delineate the effects of HFD on protein function, retinal cell physiology, and retinal disease pathogenesis [102].

While the germ-free mouse model is considered the gold-standard for microbiome studies, our conclusions are limited due to changes in baseline physiologic parameters that were altered by lack of microbiome in these mice. Retinal transcriptome changes identified in germ-free mice may be influenced by changes in immune development, metabolism, and digestion affected by the absence of microbiome.

Dietary modification is an easily modifiable risk factor, and understanding the interaction between diet, gut microbiome, and retinal disease has the potential to advance our understanding of vision-threatening diseases. Delineating these complex interactions could lead to the discovery of novel targets for intervention. While much of the focus has been on alterations to the gut microbiome as a key effector in disease pathogenesis, we present novel data suggesting that diet may affect retinal gene transcription when the microbiome is absent.

However, the microbiome-dependent and microbiome-independent effects of HFD on the retinal transcriptome remain unclear. The gut microbiome is an important mediator of the effects of diet on the retinal transcriptome, and it is currently unclear if these effects are overall protective or deleterious. Pathways in the retinal transcriptome affected by high-fat diet could be both attenuated or exacerbated by the presence of the gut microbiome, and these interactions are still poorly understood.

Despite the limitations, our study provides novel insight about potential pathways that could be involved in the diet–microbiome–retina axis and furthers our understanding of how diet may regulate disease pathogenesis and severity. Future studies are needed to define the precise role of diet in retinal diseases and to elucidate the complex, overlapping relationships in the diet–microbiome–retina axis and its involvement in retinal disease pathobiology.

## Figures and Tables

**Table 1:** Differentially expressed genes upregulated by high-fat diet (HFD).

Gene	LogFC	*p*-Value	Protein
*Fat2*	4.084	3.13 × 10^−5^	FAT atypical cadherin 2
*Npy4r*	3.518	1.10 × 10^−4^	Neuropeptide Y receptor Y4-2
*C1qtnf2*	3.248	9.57 × 10^−4^	C1q And TNF-related 2
*Deup1*	3.133	1.81 × 10^−4^	Deuterostome assembly protein 1
*Ifi204*	2.881	5.62 × 10^−3^	Interferon gamma inducible protein
*Siglec1*	2.845	3.57 × 10^−4^	Sialic acid-binding Ig-like lectin 1
*Mrap*	2.724	1.02 × 10^−3^	Melanocortin 2 receptor accessory protein
*Dmgdh*	2.672	2.89 × 10^−3^	Dimethylglycine dehydrogenase
*Maats1*	2.433	8.70 × 10^−3^	Cilia and flagella-associated protein 91
*Nppb*	2.345	4.83 × 10^−3^	Natriuretic-peptide B
*Klf1*	1.889	2.77 × 10^−4^	Kruppel-like factor 1
*Hba-a2*	1.867	7.19 × 10^−3^	Hemoglobin subunit alpha 2
*Ppp1r3g*	1.735	8.87 × 10^−3^	Protein phosphatase 1 regulatory subunit 3G
*Hbb-bs*	1.378	4.73 × 10^−3^	Hemoglobin subunit beta
*Hba-a1*	1.372	1.82 × 10^−3^	Hemoglobin subunit alpha 1
*Ms4a6b*	1.336	4.78 × 10^−3^	Membrane spanning 4-domains A6A
*Gdf2*	1.133	9.95 × 10^−3^	Growth differentiation factor 2
*Tspan11*	1.124	7.60 × 10^−3^	Tetraspanin 11
*Nlrp10*	1.103	2.63 × 10^−3^	NLR family pyrin domain containing 10

**Table 2:** Differentially expressed genes downregulated by HFD.

Gene	LogFC	*p*-Value	Protein
*Olfr690*	−3.712	8.96 × 10^−6^	Olfactory receptor family
*Gtsf1*	−3.503	1.05 × 10^−4^	Gametocyte specific factor 1
*Tcp10a*	−3.121	1.77 × 10^−3^	T-Complex 10-like 3, pseudogene
*Olfr460*	−3.077	7.60 × 10^−4^	Olfactory receptor family
*Cuzd1*	−2.902	4.51 × 10^−3^	CUB and zona pellucida-like domains 1
*Serpinc1*	−2.721	6.30 × 10^−4^	Serpin family C member 1
*Olfr691*	−2.713	1.26 × 10^−3^	Olfactory receptor family
*Opn5*	−2.708	8.80 × 10^−3^	Opsin 5
*Hist1h3i*	−2.561	5.37 × 10^−3^	H3 clustered histone 11
*Rmi2*	−2.546	6.18 × 10^−3^	RecQ mediated genome instability 2
*Rnf222*	−2.370	6.91 × 10^−3^	Ring finger protein 222
*Serpinf2*	−2.309	1.17 × 10^−3^	Serpin family F member 2
*Cstl1*	−2.302	6.08 × 10^−3^	Cystatin-like 1
*Npb*	−2.000	6.35 × 10^−3^	Neuropeptide B
*Anxa9*	−1.746	3.87 × 10^−4^	Annexin A9
*Nanos2*	−1.670	2.85 × 10^−3^	Nanos C2HC-type zinc finger 2
*Tssk4*	−1.603	8.17 × 10^−3^	Testis specific serine kinase 4
*Eqtn*	−1.510	3.93 × 10^−3^	Equatorin
*Klrg2*	−1.436	6.80 × 10^−5^	Killer-cell lectin-like receptor G2
*Mcemp1*	−1.419	3.67 × 10^−3^	Mast cell expressed membrane protein 1
*Lat*	−1.339	2.98 × 10^−3^	Linker for activation of T-cells
*Plekha4*	−1.334	7.90 × 10^−3^	Pleckstrin homology domain containing A4
*Misp*	−1.270	7.52 × 10^−3^	Mitotic spindle positioning
*P4ha3*	−1.176	4.91 × 10^−3^	Prolyl 4-hydroxylase subunit alpha 3
*Gpr84*	−1.175	9.61 × 10^−4^	G protein-coupled receptor 84
*Pi16*	−1.160	5.25 × 10^−3^	Peptidase inhibitor 16
*Slc10a5*	−1.134	4.79 × 10^−3^	Solute carrier family 10 member 5
*Ankrd7*	−1.118	7.66 × 10^−3^	Ankyrin repeat domain 7
*Tmprss5*	−1.109	2.09 × 10^−3^	Transmembrane serine protease 5
*Cdhr3*	−1.091	5.62 × 10^−3^	Cadherin-related family member 3
*Hmga2*	−1.061	1.63 × 10^−3^	High mobility group AT-hook 2
*Sycp2*	−1.061	4.34 × 10^−3^	Synaptonemal complex protein 2
*Cd27*	−1.039	5.80 × 10^−3^	T-cell activation antigen CD27
*Mypn*	−1.036	6.94 × 10^−3^	Myopalladin

## Data Availability

Complete dataset of identified DEGs is available in Supplementary Table S1.

## Supplementary Materials

The following are available online at https://www.mdpi.com/article/10.3390/cells10082119/s1, Table S1 provides all 19,681 genes selected for DEG analysis.
